# Developing an Integrated Caregiver Patient-Portal System

**DOI:** 10.3390/healthcare9020193

**Published:** 2021-02-10

**Authors:** Margaret L. Longacre, Cynthia Keleher, Marcin Chwistek, Michelle Odelberg, Mark Siemon, Molly Collins, Carolyn Y. Fang

**Affiliations:** 1 Department of Public Health, College of Health Sciences, Arcadia University, Glenside, PA 19038, USA; mfreeman_01@arcadia.edu; 2 Fox Chase Cancer Center, Web Technologies Department, Philadelphia, PA 19111, USA; Cindy.Keleher@fccc.edu (C.K.); Mark.Siemon@tuhs.temple.edu (M.S.); 3 Fox Chase Cancer Center, Supportive Oncology and Palliative Care Program, Philadelphia, PA 19111, USA; Marcin.Chwistek@fccc.edu (M.C.); Molly.Collins@fccc.edu (M.C.); 4 Fox Chase Cancer Center, Cancer Prevention and Control Program, Philadelphia, PA 19111, USA; Carolyn.Fang@fccc.edu

**Keywords:** patient–caregiver–clinician communication, family caregivers, portal system, policy

## Abstract

We have developed an integrated caregiver patient-portal system (i.e., patient–caregiver portal) that (1) allows a patient to identify their primary caregiver and their communication preferences with that caregiver in the healthcare setting; (2) connects the caregiver to a unique portal page to indicate their needs; and (3) informs the healthcare team of patient and caregiver responses to aid in integrating the caregiver. The purpose of this manuscript is to report on the formative phases (Phases I and II) of system development. Phase I involved a pre-assessment to anticipate complexity or barriers in the system design and future implementation. We used the non-adaption, abandonment, scale-up, spread, and sustainability (NASSS) framework and rubric to conduct this pre-assessment. Phase II involved exploring reactions (i.e., concerns or benefits) to the system among a small sample of stakeholders (i.e., 5 palliative oncology patients and their caregivers, *N* = 10). The purpose of these two phases was to identify system changes prior to conducting usability testing among patient/caregiver dyads in palliative oncology (phase III). Completion of the NASSS rubric highlighted potential implementation barriers, such as the non-uniformity of caregiving, disparities in portal use, and a lack of cost–benefit (value) findings in the literature. The dyads’ feedback reinforced several NASSS ratings, including the benefits of connecting caregivers and allowing for caregiver voice as well as the concerns of limited use of patient-portals by the patients (but not the caregivers) and the need for user assistance during stressful health events. One change that resulted from this analysis was ensuring that we provided research participants (users) with detailed guidance and support on how to log in and use a patient–caregiver portal. In future iterations, we will also consider allowing more than one caregiver to be included and incorporating additional strategies to enable caregivers to interact in the system as part of the care team (e.g., via email).

## 1. Introduction

Many individuals in the U.S. provide informal or family care and the demand for such care is expected to increase over the next several decades in light of a growing older adult population [1,2]. According to a national survey by the National Alliance for Caregiving (NAC) and the American Association for Retired Persons (AARP), 47.9 million individuals in the U.S. (or 19.2% of the population) were providing family care in 2019 to an adult relative or friend with disease or disability [3]. The top reasons for providing care included that a relative or friend was experiencing (as the primary condition) “old age” (16%), mobility issues (12%), Alzheimer’s/dementia (11%), surgery/wounds (6%), cancer (6%), or mental/emotional illness (5%) [3].

Though the roles and responsibilities of caregiving can vary depending upon a care recipient’s disease or disability and severity [4], common tasks across caregivers include monitoring the severity of the care recipient’s condition, managing a household, arranging care, participating in healthcare appointments, and communicating with healthcare professionals [3,5]. Because of these and other roles, the burden of caregiving is often high in terms of time, financial strain, and physical and emotional stress [5,6]. Compared to non-caregivers, caregivers are shown to utilize more health services (e.g., emergency department visits), have poorer health behaviors [7], and experience poorer overall physical [8,9] and psychosocial outcomes [4,7,8,10,11]. Furthermore, there is a small body of literature showing an association between a caregiver’s distress level and the care recipient’s level of distress [12,13]. Thus, the well-being of a caregiver is indeed important for that caregiver but also, possibly, for the care recipient as well.

Furthermore, a caregiver’s knowledge and skills pertinent to their caregiving role might also be important for care recipient outcomes, as few caregivers are fully prepared to assume the role and tasks [10,14,15]. Inadequate information among caregivers could be detrimental to care recipient outcomes [16,17]. For example, caregivers for head and neck cancer patients who needed help interacting in the clinical setting (e.g., how to talk to doctors) also reported a high need for information on reducing patient pain and distress [17].

Identifying and assisting caregivers is increasingly recognized as important in preventing or reversing adverse outcomes among caregivers as well as improving care recipient outcomes (e.g., quality of life). Given that caregivers often attend appointments and communicate with healthcare professionals [3], healthcare appointments represent an important touchpoint for assessing caregivers’ skill-related and personal needs. Yet, not all healthcare settings intentionally nor systematically involve caregivers. Indeed, in NAC’s 2016 report, entitled “Cancer Caregiving in the U.S.”, it has been reported that slightly over half (54%) of caregivers for someone with cancer had been asked by clinicians whether they needed information to care for the patient, while even fewer (29%) reported being asked if they needed information to care for themselves [10]. More recently, in NAC and AARP’s report entitled, “Caregiving in the U.S. 2020 Report,” it was highlighted that few (3 of 10) healthcare providers (e.g., doctor, nurse, or social worker) had asked the caregiver about what was needed to care for their recipient, while only 13% of caregivers indicated that a healthcare provider had asked what they need to care for themselves [3].

Calls to integrate and support caregivers in all aspects of care are evident in oncology [14,18], while the National Academies of Medicine (NAM) identified the priority of enhancing the “infrastructure” to deliver person- and family-centered care via policies and practices [19]. These recommendations to intentionally involve caregivers in healthcare settings align with state-level statutes designed to identify a caregiver in a patient’s electronic medical record and to train caregivers at hospital discharge (i.e., the Caregiver Advise, Record, Enable–C.A.R.E–Act) [20].

**Figure 1:**
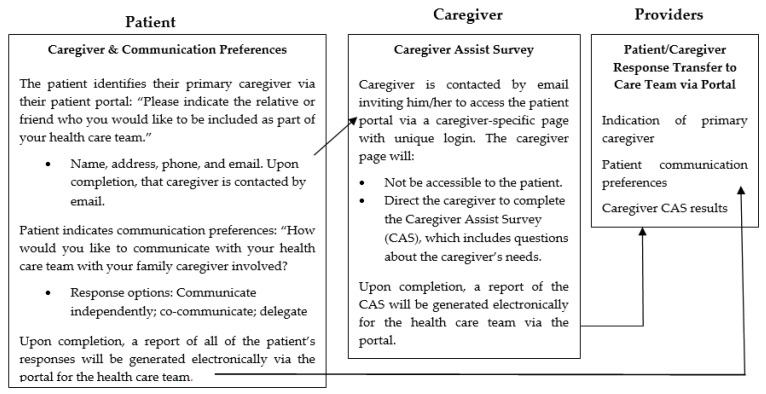
The proposed integrated caregiver patient-portal system (i.e., patient–caregiver portal).

Thus, while we understand why caregiver integration is needed and that it is recommended, much less is known about how to do this in a systematic manner. To address this need, we have developed an integrated caregiver patient-portal system (i.e., patient–caregiver portal) that (1) allows a patient to identify their primary caregiver and their communication preferences with that caregiver involved in the healthcare setting; (2) connects the caregiver to a unique portal page to indicate their needs; and (3) informs the healthcare team of patient and caregiver responses to aid in integrating the caregiver (See Figure 1). This approach does so by capitalizing on existing technology used broadly in healthcare—i.e., an existing patient-portal system.

This integrated caregiver patient-portal system is guided by several frameworks and literature. First is the concept of patient autonomy (e.g., naming a caregiver and preferences) [21,22]. Caregivers are shown to be involved in care and treatment decisions [23], while Clayman’s Autonomy framework and related published literature recognizes that caregivers can be “autonomy enhancing” or “autonomy distracting” for a care recipient/patient [24,25]. These findings and theories as well as other similar caregiving communication theories [26] suggest that understanding the caregiver–patient communication types enable health professionals to better recognize and react to patient preferences and attend to caregiver needs. Other frameworks were also foundational for the development of this integrated caregiver patient-portal system through research. Such frameworks include the National Academy of Medicine’s (NAM) Patient and Family Engaged Care Framework [27] and the American Institute for Research (AIR) Roadmap for Patient and Family Engagement in Health Care [28]. The NAM’s Framework [27] notes that those who interact with the healthcare system must participate in process change by providing feedback. Furthermore, AIR’s Roadmap [28] highlights: (1) developing procedures that specify families as members of the healthcare team; and (2) using technology to support and manage the flow of data across healthcare providers and systems (e.g., via patient portal). Tactics that support patient and family preparation include: (1) asking about priorities, experiences, and needs; (2) providing information and support; and (3) capturing patient and family satisfaction with tools and methods [28].

The purpose of this manuscript is to report on the initial phases of the development of the patient–caregiver portal, including Phases I and II. Phase I involved a pre-assessment using the non-adaption, abandonment, scale-up, spread, and sustainability (NASSS) rubric to anticipate complexity or barriers in the system design and future implementation in healthcare settings [29,30]. Phase II involved semi-structured interviewing of stakeholders (i.e., patients and their caregivers) in the palliative oncology context to explore reactions (i.e., concerns or benefits). The context of palliative oncology was selected as over 1.8 million individuals in the U.S. are expected to receive a new cancer diagnosis in 2020 and most will receive assistance from a family caregiver [31]. These two phases were initiated to assist in refining the integrated caregiver patient-portal system prior to conducting usability testing (Phase III) to be efficient with the patients’ and caregivers’ energy given the burden of cancer. Phases I and II are reported upon separately in this manuscript; however, Phase II findings are also reflected upon with respect to the NASSS assessment to note any similarities or differences.

## 2. Materials and Methods

The following describes the methodology for each of the two research phases.

### 2.1. Phase I: Potential Barriers in the Development and Implementation of an Integrated Caregiver Patient-Portal System

Phase I involved a pre-assessment using the non-adaption, abandonment, scale-up, spread, and sustainability (NASSS) rubric and literature to anticipate complexity or barriers in the system design and future implementation in healthcare settings [29,30]. The NASSS framework assists those seeking to design, develop, implement, scale-up, spread, and sustain technology-supported healthcare programs to identify key challenges prior to launching initiatives (e.g., usability testing). According to Greengalgh and colleagues [29,30], promising technological innovations in healthcare are characterized by non-adoption or abandonment by individuals or by failed attempts to scale up locally, spread distantly, or sustain the innovation long-term at the organization or system level. The NASSS framework assists in anticipating where complexity might exist for a technology project that is seeking to address a given problem and, importantly, highlights the benefits of such exploration prior to implementation in order to make adjustments proactively. The NASSS framework includes 19 questions within 7 domains, including: (1) the condition; (2) the technology; (3) the value proposition; (4) the adopter system; (5) the organization; (6) the wider context; and (7) embedding and adaptation over time. Response to domain questions is classified [30] as “simple,” “complicated,” or “complex,” and a rubric is provided for each of the classifications per question (see Results). Since this pre-assessment was not based on a specific institution but rather the concept of an integrated caregiver patient-portal system in healthcare, we excluded domains and questions specific to an institution assessment due to not being applicable. The ratings were established based on feedback from three authors (ML, CF, CK). Specifically, ML provided the first rating and guiding literature and both CF and CK reviewed independently and responded as agreeing or disagreeing. Any disagreement in rating was discussed until an agreement was reached. The final ratings are presented in Table 1, while the supporting description and literature are provided in the Results section per domain and questions.

### 2.2. Phase II: Stakeholders’ Reactions to the System

Phase II involved semi-structured interviewing of an intentionally small sample of stakeholders (i.e., patients and their caregivers) to explore reactions to the system overall and its specific components (i.e., allowing the patient to indicate a caregiver and communication preferences; allowing the caregiver to indicate needs; informing the care team of responses). This approach is similar to pretesting a survey prior to distributing it among a larger sample as well as cognitive interviewing to understand reactions [32]. Reactions were then categorized according to perceived concerns and benefits so that concerns could be discussed among the research team (including web technologies members) and addressed prior to conducting usability testing with a large sample of patient/caregiver dyads.

#### 2.2.1. Sample and Recruitment

Our sample included cancer patients who were receiving palliative care (*N* = 5) and their caregivers (*N* = 5). These patients and caregivers were recruited through the Supportive Oncology and Palliative Care Program (SOPC) at Fox Chase Cancer Center. The study was explained to both the patient and their caregiver by a study Research Assistant or SOPC staff. The study was approved by Fox Chase Cancer Center’s Institutional Review Board, and each patient and caregiver provided individual written informed consent prior to participation.

#### 2.2.2. Procedures and Measurement

Following consent, the palliative oncology patient/caregivers dyads were invited to watch a 4-min video describing the system and components (see Figure 1). Following a review of the video, the patients and caregivers participated in semi-structured interviews with the study Research Assistant. All interviews were audio-recorded with participant permission. To elucidate benefits and concerns, the participants were asked open-ended questions about the helpfulness of the system overall and specific components and what was liked and disliked. The caregivers were also asked if they would like to receive information and support in any of the following ways: In-person with the care team; by telephone with the care team; being directed to Internet resources; being provided with tailored information sent via the patient portal/caregiver-specific page (yes or no for each).

#### 2.2.3. Analysis

To determine benefits and concerns, qualitative analyses were performed for the stakeholders’ responses to the open-ended questions about system helpfulness and aspects liked or disliked. Specifically, the first author (ML) checked all transcripts for accuracy by reviewing the transcripts while listening to the audio recordings. Qualitative analysis of the patient and caregiver transcripts utilized an integrated [33,34] (deductive and inductive) approach to analysis, which involved indicating comments as either a benefit or concern along with a description (i.e., subtheme or recurrent concept or statement). This integrated qualitative approach for analysis is effective and efficient when seeking a defined purpose (e.g., noting concerns to discuss with the Web Technologies Department) [33,34].

To analyze the interviews, ML read through the transcripts for each of the stakeholder groups to gain an overall understanding and then coded text (comments) as either a benefit or concern. Next, text identified as a benefit or concern was further described (i.e., proposed subthemes) to give more depth and understanding and to compare across transcripts. These subthemes were then compared for two of the coded transcripts per stakeholder group to establish a codebook. This codebook was to be used for additional coding by ML and for secondary coding by an additional author (MO) for an independent assessment of the transcripts. After MO coded two transcripts, ML and MO discussed any concerns with the codebook and made a minor adjustment, including combining two subthemes into one and modifying the description of a subtheme to be concrete. The benefit and concern subthemes for the patients and caregivers are provided in Table 2 and Table 3, respectively. Coding was conducted using NVivo 12 in order to assess inter-rater reliability with the kappa coefficient calculated by the software. Kappa coefficients indicated excellent inter-rater reliability for both patient and caregiver assessments for the benefit and concern subtheme coding, including a kappa of 0.914 for coding agreement of the caregiver transcripts and 0.889 for the patient transcripts. A kappa coefficient of 0.60–0.79 indicates moderate agreement and values exceeding 0.80 indicate excellent agreement [35]. The primary goal was to identify consistent concerns regarding the system prior to usability testing among a large sample of palliative oncology patient/caregiver dyads. “Consistent concerns” was operationalized as three or more individuals per stakeholder group noting a similar concern. Consistent concerns were discussed with the research team, including web technologies experts, to explore possible changes prior to usability testing and into the future. Frequencies were also conducted to report the caregivers’ preferences for receiving information and support in specific ways.

## 3. Results

### 3.1. Phase I: Potential Barriers in the Development and Implementation of an Integrated Caregiver Patient-Portal System

Ratings to questions per the NASSS rubric are provided in Table 1, while further description and supporting literature are provided in the text below per domain and question. In sum, eight questions were rated as “complicated” and five rated as “simple.” We did not rate all elements of the “Organization” domain as our assessment pertains to the concept of the system broadly in healthcare and not institution-specific implementation, given this tool is conceptualized to be used in varied healthcare settings in which a patient-portal system exists.

**Table 1:** Non-adaption, abandonment, scale-up, spread, and sustainability (NASSS) framework rubric rating.

	Rating		
Domain and Questions	Simple	Complicated	Complex
**The Condition or Illness**			
What is the nature of the condition?	Well-characterized, well-understood, predictable	Not fully characterized, understood, or predictable	Poorly characterized, poorly understood, unpredictable, or high risk
What are the relevant socio-cultural factors and comorbidities?	Unlikely to affect care significantly	Must be factored into care plan and service model	Pose significant challenges to care planning and service provision
**The Technology**			
What are the key features of the technology?	Off-the-shelf or already installed, freestanding, dependable	Not yet developed or fully interoperable; not 100% dependable	Requires close embedding in complex technical systems; significant dependability issues
What kind of knowledge does the technology bring into play?	Directly and transparently measures [changes in] the condition.	Partially and indirectly measures [changes in] the condition	Link between data generated and [changes in] the condition is currently unpredictable or contested
What knowledge and/or support is required to use the technology?	None or a simple set of instructions	Detailed instruction and training needed, perhaps with ongoing helpdesk support	Effective use of technology requires advanced training and/or support to adjust to new identity or organizational role
What is the technology supply model?	Generic, “plug and play”, or COTS solutions requiring minimal customization, easily substitutable if supplier withdraws	COTS solutions requiring significant customization or bespoke solutions; substitution difficult if suppliers withdraw	Solutions requiring significant organizational reconfiguration or medium-to-large scale-bespoke solutions, highly vulnerable to supplier withdraw
**The Value Proposition**			
What is the developer’s business case for the technology (supply-side value)?	Clear business case with strong chance of return on investment.	Business case underdeveloped; potential risk to investors	Business case implausible; significant risk to investors
What is its desirability, efficacy, safety, and cost-effectiveness (demand-side value)?	Technology is desirable for patients, effective, safe, and cost-effective.	Technology’s desirability, efficacy, safety, or cost-effectiveness is unknown or contested	
**The Adopter System**			
What changes in staff roles, practices, and identities are implied?	None	Existing staff must learn new skills and/or new staff be appointed.	Threat to professional identity, values, or scope of practice, risk of job loss
What is expected of the patient (and/or immediate caregiver)—and is this achievable by, and acceptable to, them?	Nothing	Routine task, e.g., log on, enter data, converse	Complex tasks, e.g., initiate changes in therapy, make judgments, organize
What is assumed about the extended network of lay caregivers?	None	Assumes caregiver will be available when needed	Assumes a network of caregivers with the ability to coordinate their input
**The Wider Context**			
What is the political, economic, regulatory, professional, and sociocultural context for program rollout	Financial and regulatory requirements already in place nationally; professional bodies and civil society supportive	Financial and regulatory requirements being negotiated nationally; professional and lay stakeholders not yet committed	Financial and regulatory requirements raise tricky legal or other challenges; professional bodies and lay stakeholders unsupportive or opposed
**Embedding and Adaptation Over Time**			
How much scope is there for adapting and coevolving the technology and the service over time?”	Strong scope for adapting and embedding the technology as local need or context changes	Potential for adapting and coevolving the technology and service is limited or uncertain	Significant barriers to further adaptation and/or coevolution of the technology or service

NASSS framework rubric rating for the integrated-caregiver portal system is indicated in bold with grey highlight for the domains and questions assessed (the full rubric is available in [30]. “The Organization” domain questions and question #2 of “Embedding and Adaptation Over Time” domain were not included in the table because they were not rated as this pre-assessment was not based on a specific institution but rather the concept of patient–caregiver portal in healthcare broadly.

#### 3.1.1. The Condition

The Condition domain considers the clinical condition as well as the comorbidities and sociocultural aspects of a condition via two questions. As noted in Table 1, the first question in this domain is “What is the nature of the condition or illness?” In utilizing the NASSS framework rubric, we suggest that caregiving is “complicated” as it is “not fully characterized, understood, or predictable.” Indeed, reasons for providing family care are varied, and because of this, the demands of care and related roles or tasks often differ. For example, a caregiver for someone with head and neck cancer might be more likely to monitor the patient’s eating, swallowing, and nutrition compared to other cancer contexts (e.g., breast cancer) [36,37]. A caregiver for someone with advanced dementia will also commonly assist with medical nursing tasks, including management of medications and nutritional intake and supplementation [38], but such a caregiver might also manage a care recipient’s “behavioral crises” such as wandering or agitation [39]. Moreover, caregivers are expected to perform many roles in patient care, including monitoring symptoms and performing medical/nursing tasks [1,10], often with little preparation [10,14,15]. Thus, the development of information and training for caregivers—which is a future component of this system—likely involves a combination of general or base-level resources for all caregivers as well as disease or care context-specific content.

We also categorize the second question, “What are the relevant sociocultural factors and comorbidities?” as “complicated.” Socio-cultural factors are inherent within caregiving. For example, clinicians must consider a patient’s autonomy in making care and treatment decisions, but there can also be many interpersonal and socio-cultural beliefs and values that not only shape a patient’s experience and decision-making but also that of the caregiver(s) (e.g., a caregiver’s choice in being a caregiver [40,41] or family hierarchy and traditional cultural care roles within families and society [42]. A key component of the system as proposed is to understand or alleviate socio-cultural concerns by allowing a patient to name a preferred or primary caregiver and indicating how he/she prefers to communicate when that caregiver is involved.

#### 3.1.2. The Technology

This domain considers the material and technical features of a proposed technology via four questions. We rated the first question “What are the key features of the technology?” as “complicated” (i.e., “not yet developed or fully interoperable”; see Table 1). The technology, a traditional patient-portal system, was expanded to include a patient’s preferred caregiver, to assess the caregiver’s information needs, and to communicate patient/caregiver dyad responses back to the care team electronically. Thus, the complexity refers to the process of connecting the caregiver page and functionality of information transfer, and that this system would then need to be replicated for each unique patient portal system when disseminated to other institutions.

Other aspects within this domain, however, are rated as “simple.” For example, we rated question two as “simple”, because the system’s caregiver questions will allow for the measurement of caregiver needs, and thus it does “directly and transparently measure the condition/need” per the rubric. However, measuring caregiver needs cannot be disconnected from the broader complexity of meeting caregiver needs within a given institution. Meeting caregiver needs is shown to be limited by resources, designated staff, and a lack of healthcare system reimbursement for time spent with caregivers [43,44]. The third question refers to the knowledge needed by the user, “What knowledge and/or support is required to use the technology?” Here, we also provide a complexity rating of “simple” (i.e., “None or a simple set of instructions”). Use of patient portals is shown to vary by race, ethnicity, and age and, thus, should be monitored as part of the ongoing research and implementation process [45]. Lastly, with respect to the technology supply side, the system does require some customization but likely can be replicated and adapted in other portals.

#### 3.1.3. The Value Proposition

The Value Proposition domain pertains to whether a new technology is worth developing and for whom it generates value and is considered via two questions, including: “What is the developer’s business case for the technology (supply-side value)?” and “What is its desirability, efficacy, safety, and cost-effectiveness (demand-side value)?” We rated the complexity for these value proposition questions as “simple” and “complicated”, respectively (see Table 1). In terms of supply-side value, research has shown that caregivers are receptive to information and support programming and that such programs benefit caregivers as well as patients [46,47]. If system outcomes, including improvements and quality of care or reductions in unintended service use (e.g., rehospitalization), are identified via subsequent research, the supply-side—meaning a hospital or system uptake—will be greater. Early research in Alzheimer’s disease showed that a psychosocial intervention for caregivers was associated with delays in nursing home care placements among care recipients [48,49].

#### 3.1.4. The Adopter System

This domain refers to complexity from the lens of professional staff, patients, and lay caregivers and involves three questions (see Table 1). The first two questions regarding staff roles and patient/caregiver expectations are rated as “complicated”. We are designing the system to be embedded within the flow of current systems for use in outpatient care and to connect and make information accessible. Our subsequent user testing will also explore clinical benefit from the perspective of clinicians, and it is likely that perceptions of benefit will depend upon broad acceptance and perceived value by all clinicians and staff. The AIR Roadmap for Patient and Family Engagement in Health Care [28] as well as NAM’s Patient and Family Engaged Care Framework [27] both note that clinician leadership is key in successfully engaging and communicating with caregivers.

Furthermore, our user testing will also allow us to quantify “achievement” by patients and caregivers in terms of actual use. We anticipate that varied factors might impact use, such as time demands, technology skill demands, and distress as was demonstrated in previous literature related to patient portal use among patients [50,51]. Finally, given that the focus of this system is to deliver patient- and family-engaged care, we suggest little to no assumptions would be made about the caregiver, and in fact, the point of the system is to reduce clinicians’ assumptions about caregiver involvement and patient preferences.

#### 3.1.5. The Organization

The Organization domain refers to an organization’s capacity and readiness to adopt system change—the engagement of caregivers within the flow of care in a systematic way. We did not provide ratings for a specific organization as the implementation of the system will be organization and health-system dependent. Our current purpose was not to directly assess a specific organization’s capacity and readiness; rather, the current need is to consider the dissemination and implementation of this system broadly within the health care field (which will include specific follow-on studies in varied healthcare settings). Currently, the degree of caregiver integration varies greatly across institutions and among clinicians, which speaks to high complexity when considering engagement universally across hospitals and systems. According to NAM’s Patient and Family Engaged Care Framework [27], engagement of caregivers in clinical care is dependent upon clinician skill and training (as noted above), but it is also dependent upon internal organizational foundations, including commitment and policies related to patient- and family-engaged care, and current structures or practices that enable or limit it.

#### 3.1.6. The Wider Context

This domain refers to the wider institutional or societal context and involves consideration of the following question: “What is the political, economic, regulatory, professional, and sociocultural context for program rollout?” This domain is rated as “complex” as there are many health system barriers to engaging caregivers in clinical care as noted above. However, a systems culture toward patient- and family-engaged care is increasing and being led, importantly, by policy—e.g., the Caregiver Advise Record Enable (C.A.R.E) Act developed by AARP and now implemented in some form in over 40 U.S. states and territories [20]. Moreover, the emphasis on integrating caregivers in oncology also continues to strengthen [14] echoing other disease contexts in which caregivers are involved in clinical care as a means to elicit and prioritize goals of care (e.g., dementia; older adult care) [52,53]. The Institute of Medicine (IOM) has long voiced the need to appropriately involve caregivers in care for older adults [1,52], while, recently, the National Academy of Sciences stressed the need to enhance the “infrastructure”, via policies and practices, to deliver person-and-family centered care [19]. Thus, ongoing advancement in policy is vital to the successful dissemination of this system to broaden caregiver engagement and integration.

#### 3.1.7. Embedding and Adaptation Over Time

This domain refers to the future adaptation of the system, and complexity is explored via two questions: “How much scope is there for adapting and coevolving the technology and the service over time?” and “How resilient is the organization to handling critical events and adapting to unforeseen eventualities?” The first question is rated as “complicated.” There is the capacity to adapt the system to varied institutions; however, adapting the technology to provide caregiver access to a portal is not currently part of the institutions’ portal setup, and to integrate the electronic delivery of results to the health care team into existing workflow and systems is complex. Moreover, the system itself can also be adapted to modify questions as needed to fit specific settings. The resiliency of organizations will need to be explored via future research in which we might test the system in diverse settings. However, this stage is in the future and thus difficult to fully anticipate concerns as the broader system engagement of caregivers might be varied from that of today.

### 3.2. Phase II: Stakeholders’ Reactions to the System

#### 3.2.1. Sample Characteristics

The patients (*N* = 5) were 57.8 years of age on average (range: 53–69), and predominantly male (60%), white (80%), and non-Hispanic (100%). The cancer types of the patients varied (i.e., melanoma; renal cell carcinoma; lung cancer; hepatocellular carcinoma, and breast cancer), and all patients had advanced disease, with cancer stage ranging from III (B) to IV. The average number of months from diagnosis to the interview date was 27 (2.25 years; Range: 1 month–108 months). The majority (80%) of patients were 12 months or less from their diagnosis date at the time of the interview. The caregivers (*N* = 5) were 61.2 years of age on average (range: 49–80) and predominately female (60%), white (80%), and non-Hispanic (100%).

#### 3.2.2. Concerns and Benefits

Table 2 and Table 3 highlight the benefits and concerns noted by the patients and caregivers, respectively, and includes exemplar quotes. The most common benefit of the system among patients was that it connects the caregiver into care (Connects Caregiver) (*n* = 4) and allows for expression about caregiver barriers (Learn Caregiver Barriers) (*n* = 4) (see Table 2). The most common concern among patients was not using the portal (Lack of Portal Use) (*n* = 3). For example, three of the five patients noted not using the portal, but two of these patients noted that their caregiver was comfortable with portal use.

**Table 2:** Benefit subthemes, descriptions, and exemplars for patients and caregivers.

Subtheme	Description	# Of Patients Referencing	Patient Exemplar Quote	# Of Caregivers Referencing	Caregiver Exemplar Quote
Connects Caregiver	Identifies and connects caregiver with the patient’s care team	4	*I like the caregivers to be totally involved.*	5	*[I like that] It identifies a caregiver.*
Gives Voice	Allows caregiver to express needs	2	*She may have questions of her own that she really doesn’t want to talk to me about, she can you know go [to the system] because it’s stressful on her and she might want to say something*	4	*There may be things that I am thinking that I may not want to put stress on him about then I can ask questions to and receive answers back*
Learn Caregiver Barriers	Helps the care team recognize barriers, feelings, or needs related to caregiving	4	*When you fill out the forms they [care team] kind of get to know you and you know one of the questions may be how comfortable are you [providing care] just like you asked [in the demonstration] … or questions like how do you feel doing this.*	2	*You can get the support needed and without guessing or trying to figure out who you need to go to—people [care team] will see it and recommend what you need.*
Patient Autonomy	Considers or improves patient autonomy	3	*Well it’s ultimately up to the patient to decide how he really wants or she wants her, their case managed—and how much help they want from the caregiver and you can’t do it without that input.*	0	(Not referenced by caregivers)
Ease of Use of Integrated Portal System	Perception that the integrated portal system is easy to use or beneficial than standard of care	3	*Seemed pretty straight forward.*	4	*It looked okay to me. I am sure it’s doable for someone who’s used to doing the portal if you can do the patient portal then it should be okay to do this.*
Portal Use Among Patient	Currently or previously comfortable using the patient portal	3	The patient portal is a great feature for those who are capable enough to use it.	0	(Not referenced by caregivers)
Simplifies Communication	Simplifies communication with care team	1	*It seems to be easier than trying you know to explain everything over again and like I said I might forget something; she might have a question instead of asking me she can just go [to the system].*	3	*It gets you involved, you know when the appointments are, what the treatment plans are, you can help interpret everything for her and know for sure what is going on and not just hear it or be told second hand what is going on.*
Privacy	Protects patient or caregiver privacy within the system	0	(Not referenced by patients)	2	*I think the easy use of the system if he has, he can limit what I need to see or what I’m allowed to use on the system which is good but it’s also good because I think it’s important for the caregiver to have that access with the team.*
Avoids Assumptions	Avoids making assumptions about who the caregiver is for the patient	0	(Not referenced by patients)	3	*It communicates how much she wants me to be involved.*

Like patients, caregivers perceived that the system would improve the ability to connect the caregiver into the healthcare setting (Connects Caregiver) (*n* = 5), followed by the perceived benefit of giving the caregiver an active voice in care conversations (Gives Voice) (*n* = 4). The most common concern noted among caregivers pertained to the adaptability of the system, such as whether the system could accommodate more than one caregiver or allow a change in the primary caregiver (Adaptability) (*n* = 4), wanting more interactive features, such as chatting (Expectations) (*n* = 3), and need for hands-on testing (Need for User Testing) (*n* = 3) (see exemplar quotes in Table 3).

**Table 3:** Concern subthemes, descriptions, and exemplars for patients and caregivers.

Subtheme	Description	# of Patients Referencing	Patient Exemplar Quote	# of Caregivers Referencing	Caregiver Exemplar Quote
Adaptability	Suggested additions/changes to system now or in future	0	(Not referenced by patient)	4	*Letting there be more than one person [as caregiver].*
Expectations	Expectations for the system not aligning with beta functioning	1	*Integration with… asking a live (question), not that it’s urgent but get back to me, or whatever…*	3	*Yeah, because then I don’t have to place the phone call I can type it in—hey, I got a question about this, or can somebody contact me—you know I can just put it in the system and it will go through to his care team.*
Need for User Testing	Need to use the system “hands-on”	1	*I would have to use it a few times.*	3	*I still think it should be rolled out and then used.*
Overwhelming	Cancer treatment and diagnosis involves high volume of information	1	*I think somebody needs to walk them through it… when you get in there you’re in shock.*	1	*I [caregiver has a nursing background] have an edge above the typical lay person who’s coming in here in a whirlwind of emotions and what the heck do we do next… oh my god it’s information overload.*
Lack of Portal Use	Patient does not use the current portal system or have previous experience	3	*I never used it.* *She [caregiver] is very computer literate and she has no problem at all going on there you know.*	0	(Not referenced by a caregiver)

As part of the interview, caregivers were asked about preferences for receiving information and support. Four caregivers indicated that receiving information and support in-person would be helpful, but one caregiver noted it would depend on the day in terms of wanting to talk. All caregivers indicated that receiving information or support by telephone would be helpful. Three caregivers indicated that being directed to the Internet would be helpful, while all the caregivers indicated that being provided with tailored information or resources made available via the patient–caregiver portal caregiver-specific page would be helpful.

## 4. Discussion

Consideration of the NASSS framework allowed us to identify potential barriers to implementing this patient–caregiver portal system broadly in healthcare settings. Some of the barriers are amendable immediately while other concerns will require adaptation over time. For example, ratings of “complicated” were most evident in the “condition” domain in reference to caregiving. Although caregiving is a general concept, the act of caregiving varies according to the disease or disability of the care recipient. According to the NAC/AARP Caregiving in the U.S. report [5], certain caregiving experiences are higher in burden and require more information and support. Information is often needed to be disease-specific, particularly in the case of cancer, given that the cancer site, disease stage, and treatment regimen all have important implications for the care needed. Caregiving is also complicated in light of many socio-cultural considerations, and, thus, the system will likely need to incorporate caregiving-related cultural competence training for institutions and clinicians. We are in the process of developing a library to synthesize existing caregiving resources to inform care teams of available resources, and this is a needed area of development in the field with respect to understanding cultural competence in the caregiving context.

This exercise in anticipating barriers also reinforced the importance of our future user testing and how caregiver needs are addressed. We reflect in The Technology domain assessment that the process of asking questions—assessing caregiver needs—is “simple” from a research and technology perspective. However, from an implementation perspective, there are possible barriers that could move the rating higher in terms of complexity if institutions are not adequately resourced with personnel or financially to respond to caregiver needs. To adequately meet needs, there must be collaboration within and across institutions and entities (e.g., non-profit organizations) [43]. This collaboration might be institution-dependent, but as noted above with the development of a resource library, we will also be looking to provide recommended collaboration tips and referrals. Some recommendations and strategies to enhance patient- and family-engaged care are available and can be helpful for institutions looking to implement such care [27,28].

Similarly, this pre-assessment shed light on the unknown immediate and long-term financial value (cost–benefit analysis) of the system. Such assessment is a future goal for this work, but, to date, comparisons in the literature are lacking. Indeed, quantifying the benefit of programming in financial terms and quality of life terms is greatly needed. Related to this need is an ongoing assessment of the impact on staff time and resources, and the ability of staff to be educated and inform caregivers or refer to external community groups and related spending and savings. The potential to triage needs is noted by Alfano and colleagues in the context of addressing the needs of cancer caregivers [18]. As noted, we anticipate using the system to relay and refer to currently available caregiving resources (e.g., online and in the community). Although the long-term financial benefit remains unknown, the exploration of such is in line with current funding opportunities, which demonstrates the prioritization of such assessments in the future.

Using the NASSS framework also allowed for consideration of the importance of organizational and broader healthcare system culture for system implementation. This system is potentially a step toward understanding how to engage caregivers, but this step must be concurrent with processes within organizations and, importantly, within the healthcare system to enable clinicians, institutions, and insurers to engage caregivers (e.g., reimbursement of provider time). Indeed, as noted by Greenhalgh and colleagues [30], the “wider context” domain is often key to explaining an organization’s failure to move from a successful demonstration project to a fully mainstreamed service that is transferable to other healthcare settings and is sustainable.

The stakeholders’ feedback (Phase II) reinforced several of the Phase I NASSS ratings. For example, with respect to the Value Proposition domain, both patients and caregivers most commonly noted the benefit of connecting the caregiver into healthcare. Similarly, patients also indicated the benefit of learning the caregivers’ barriers to providing care. For caregivers, the system was viewed as giving voice for caregivers to indicate concerns without alerting the patient or in a manner that would “not put stress on him.” This feedback suggests that the system would be of high value for these patients and caregivers. In addition, patients also referenced patient autonomy or the ability to identify the caregiver of choice and how they want to communicate with that caregiver. Caregivers noted more often than patients that the tool might help to simplify communication (e.g., not repeating oneself over and over to each clinician), and all were receptive to receiving tailored information through the system.

Data obtained from the patient and caregiver interviews also highlighted unique concerns to be addressed prior to broader testing in oncology. For example, several patients shared that they did not use patient-portals themselves but that their caregivers were more comfortable using such systems. The use of patient-portals is shown to vary by race, ethnicity, and age [45], and our next phase of usability testing will explore the use of this patient–caregiver portal among a large sample of dyads and assess differences by sociodemographic characteristics. Furthermore, one dyad pointed out that a cancer diagnosis is overwhelming in terms of emotions and information, and, thus, navigating a system can be difficult. These barriers suggest a need to be proactive in providing assistance to users. As a result, technical assistance for users will be incorporated during the usability testing phase. Additional important recommendations included being able to incorporate multiple caregivers or caregiving teams without detracting from patient autonomy and varied ways to interact with the healthcare team. In future iterations, we will consider allowing more than one caregiver to be included and incorporating additional strategies to enable caregivers to interact in the system as part of the care team (e.g., via email).

## 5. Conclusions

Both phases allowed us to identify ways to modify the system prior to user testing and inform the long-term development and implementation of the system in oncology and beyond. It is important to note that the number of patients and caregivers interviewed for stakeholder feedback was low; however, the goal was not to reach saturation of themes but to explore stakeholders’ reactions to the prototype system prior to the next phase of usability testing. One change that resulted from this analysis was ensuring that we provided research participants (users) with detailed guidance and support on how to log in and use a patient–caregiver portal and to provide support as needed given the emotional strain of a serious health event. We will also consider allowing more than one caregiver to be included and incorporating additional strategies to enable caregivers to interact in the system as part of the care team (e.g., via email). We believe this two-phase assessment provided important first steps in developing and implementing a unique patient–caregiver portal system for healthcare toward the benefit of caregivers and their care recipients.

## Data Availability

The data are not stored in a publicly archived repository due to privacy concerns. De-identified data can be made available by contacting the first author.
